# Immunomodulatory and Antiviral Activity of Metformin and Its Potential Implications in Treating Coronavirus Disease 2019 and Lung Injury

**DOI:** 10.3389/fimmu.2020.02056

**Published:** 2020-08-18

**Authors:** Xianyang Chen, Huifang Guo, Li Qiu, Chengdong Zhang, Qiang Deng, Qibin Leng

**Affiliations:** ^1^Affiliated Cancer Hospital & Institute of Guangzhou Medical University, State Key Laboratory of Respiratory Diseases, Guangzhou Medical University, Guangzhou, China; ^2^Department of Clinical Oncology, Taihe Hospital, Hubei University of Medicine, Shiyan, China; ^3^Department of Microbiology and Parasitology, School of Basic Medical Sciences, Shanghai Medical College, Fudan University, Shanghai, China

**Keywords:** coronavirus disease 2019, SARS-CoV-2, cytokine storm, metformin, antiviral activity

## Abstract

The pandemic of coronavirus disease 2019 (COVID-19), a disease which causes severe lung injury and multiple organ damage, presents an urgent need for new drugs. The case severity and fatality of COVID-19 are associated with excessive inflammation, namely, a cytokine storm. Metformin, a widely used drug to treat type 2 diabetes (T2D) mellitus and metabolic syndrome, has immunomodulatory activity that reduces the production of proinflammatory cytokines using macrophages and causes the formation of neutrophil extracellular traps (NETs). Metformin also inhibits the cytokine production of pathogenic Th1 and Th17 cells. Importantly, treatment with metformin alleviates various lung injuries in preclinical animal models. In addition, a recent proteomic study revealed that metformin has the potential to directly inhibit SARS-CoV-2 infection. Furthermore, retrospective clinical studies have revealed that metformin treatment reduces the mortality of T2D with COVID-19. Therefore, metformin has the potential to be repurposed to treat patients with COVID-19 at risk of developing severe illness. This review summarizes the immune pathogenesis of SARS-CoV-2 and addresses the effects of metformin on inhibiting cytokine storms and preventing SARS-CoV-2 infection, as well as its side effects.

## Introduction

In recent decades, the world has experienced outbreaks of newly emerging viruses, including severe acute respiratory syndrome coronavirus (SARS-CoV-1), Middle East respiratory syndrome coronavirus (MERS-CoV), H5N1 virus, pandemic H1N1 virus, and H7N9 virus (1–4). Currently, coronavirus disease 2019 (COVID-19), caused by SARS-CoV-2 infection, has become a global pandemic. Similar to the H7N9 virus (5, 6), SARS-CoV-2 causes high mortality in elderly patients who have preexisting chronic diseases. Acute lung injury is one of the major causes of the high mortality of patients infected with H5N1, H7N9, MERS-CoV, SARS-CoV-1, and SARS-CoV-2 (1–4). Rising evidence suggests that an elevated inflammatory immune response, characteristic of cytokine storms (7–10), is linked to the acute lung injury and fatality caused by these viruses (11–13).

To date, there is no effective treatment for acute lung injury caused by viral infections. Metformin is a clinically approved anti-diabetes drug. Recent studies have shown that metformin not only has immunomodulatory and antiviral activities but also prevents various acute lung injuries in animal models (14, 15). Thus, this article reviews the immune pathogenesis of SARS-CoV-2 infection and discusses the possibility of using metformin as a drug for mitigating COVID-19 illness severity.

## Excessive Inflammation Likely Contributes to the Severe Illness of Patients With COVID-19

SARS-CoV-2 infects human epithelial cells by binding to human angiotensin-converting enzyme 2 (ACE2). Single-cell sequencing analysis shows that the *ACE2* gene is expressed in cell clusters in organs including the lung, heart, esophagus, kidney, bladder, testis, and ileum (16–18), indicating that these organs are at risk of SARS-CoV-2 infection. Consistently, multiorgan failure has been observed in some patients with severe COVID-19 (7, 10, 19). The ACE2 expression profile suggests that SARS-CoV-2 infection may initiate or even directly cause organ failure (20).

In addition to direct infection, evidence shows that an elevated inflammatory immune response is also involved in the pathogenesis of SARS-CoV-2 infection. Patients in the intensive care unit (ICU) had higher plasma levels of IL-2, IL-7, IL-10, G-CSF, IP-10, MCP-1, MIP-1α, and TNFα than non-ICU patients (7). Some severe patients also have elevated IL-6 levels in their plasma (21, 22), which correlates with respiratory failure and fatality (19, 22). In addition, the serum levels of C-reactive protein, which is positively regulated by IL-6, are elevated in patients with severe COVID-19 (7, 22). The systemic elevation of cytokines is reminiscent of the cytokine storm or cytokine release syndrome (CRS) that occurs in severe infections of influenza viruses, MERS-CoV, and SARS-CoV-1 (20, 23). Due to the critical role of IL-6 in promoting CRS, inhibition of IL-6 or IL-6 receptor with antibodies has been proposed to treat patients with severe COVID-19 (23, 24).

How SARS-CoV-2 infection triggers cytokine storms remains largely speculative. Concomitant with elevated cytokines, patients with severe COVID-19 exhibit marked lymphopenia. A recent autopsy study of patients with COVID-19 shows that SARS-CoV-2 infection is detected in CD169^+^ macrophages that express the ACE2 molecule and produce IL-6. The infection of these CD169^+^ macrophages correlated with lymphocyte apoptosis in the spleen and lymph nodes. This finding may partly explain the excessive inflammation and lymphopenia during severe SARS-CoV-2 infection (25).

Consistent with the involvement of excessive innate immunity in the pathogenesis of severe COVID-19, infiltration of neutrophils in the lung has been found in an autopsy specimen (26). High neutrophil counts are significantly associated with COVID-19 fatality (10), and the neutrophil-to-lymphocyte ratio is an independent risk factor for the illness severity of COVID-19 (27). When a cytokine storm occurs, chemokines recruit neutrophils to lung lesions, and proinflammatory cytokines, such as IL-1β and IL-6, activate neutrophils to produce NETs, which may contribute to lung damage and mortality in patients with COVID-19 (26). It has been reported that aberrant NET formation is linked to pulmonary diseases, particularly acute respiratory distress syndrome (26). Therefore, targeting NETs with existing drugs is also proposed to reduce the clinical severity of COVID-19 (26).

**FIGURE 1:**
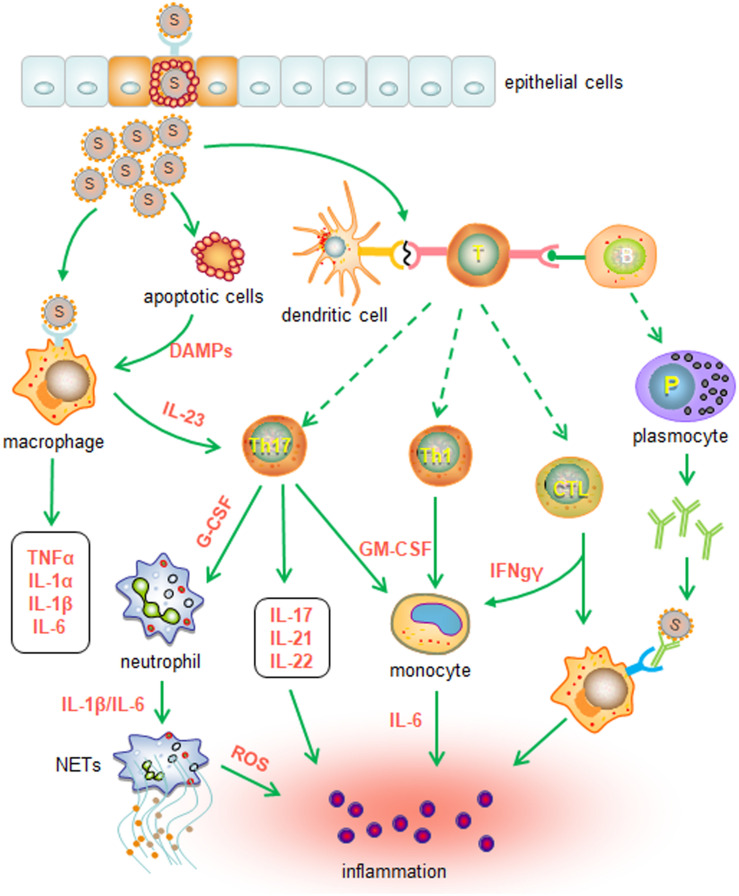
Putative innate immune responses induced by SARS-CoV-2 infection. SARS-CoV-2 and DAMPs released from damaged cells activate macrophages and cause the production of proinflammatory cytokines, such as IL-1α, IL-1β, IL-6, and TNFα, in the early stage of infection. The adaptive immune responses subsequently lead to the secretion of proinflammatory cytokines and chemokines, which may promote further innate cell recruitment and activation. Furthermore, activated Th17 cells and Th1 cells may recruit neutrophils and monocytes through the release of G-CSF and GM-CSF, respectively. Activated CTLs may drive the differentiation of monocytes and macrophages by secreting IFNγ. Inflammatory chemokines and cytokines also recruit and activate neutrophils to produce ROS and NETs. Taken together, the cytokine storm induced by innate immune cells ultimately results in inflammation and injury.

Several pieces of evidence suggest that aberrant activation of immune cells may also contribute to cytokine storms and pathogenic manifestations in the lungs. Single-cell analysis of bronchoalveolar lavage fluid shows that patients with mild COVID-19 have an expansion of clonal CD8 T cells, whereas patients with severe disease have a decline in T cells and NK cells and a concomitant increase in inflammatory FCN1^+^ macrophages (28). These observations suggest that weak adaptive immunity may result in an inadequate ability to control viral infection. Thus, the persistent presence of SARS-CoV-2 activates alveolar macrophages or epithelial cells to produce various proinflammatory cytokines and chemokines, triggering or recruiting more innate immune cells and thereby amplifying inflammation (20, 29). Furthermore, aberrant Th1 and Th17 cells may also promote the activation of innate immune cells by producing proinflammatory cytokines, including IFNγ, IL-17, and TNFα (13, 24, 30). In addition, damage-associated molecular patterns (DAMPs) released by injured lung epithelial cells could activate innate immune cells as well. The cascade of innate immune responses ultimately results in uncontrolled inflammation (20) (Figure 1).

## Metformin Inhibits Inflammatory Responses and Alleviates Acute Lung Injuries

Metformin, namely, N,N-dimethylbiguanide, is a first-line drug to treat type 2 diabetes (T2D) and metabolic syndromes. Metformin has a high safety profile and is thus widely used. Its glucose-lowering effect is efficacious when used as monotherapy or in combination with other antidiabetic agents. Patients with T2D who are treated with metformin also had a significant reduction in complications, including myocardial infarction, hypertrophy, and diabetic cardiomyopathy, suggesting that metformin has a cardiovascular protective effect (31). In addition, recent studies have revealed that metformin has numerous other beneficial effects for underlying diseases, including anticancer, anti-aging, neuroprotective, and immunomodulatory effects (31–33).

**FIGURE 2:**
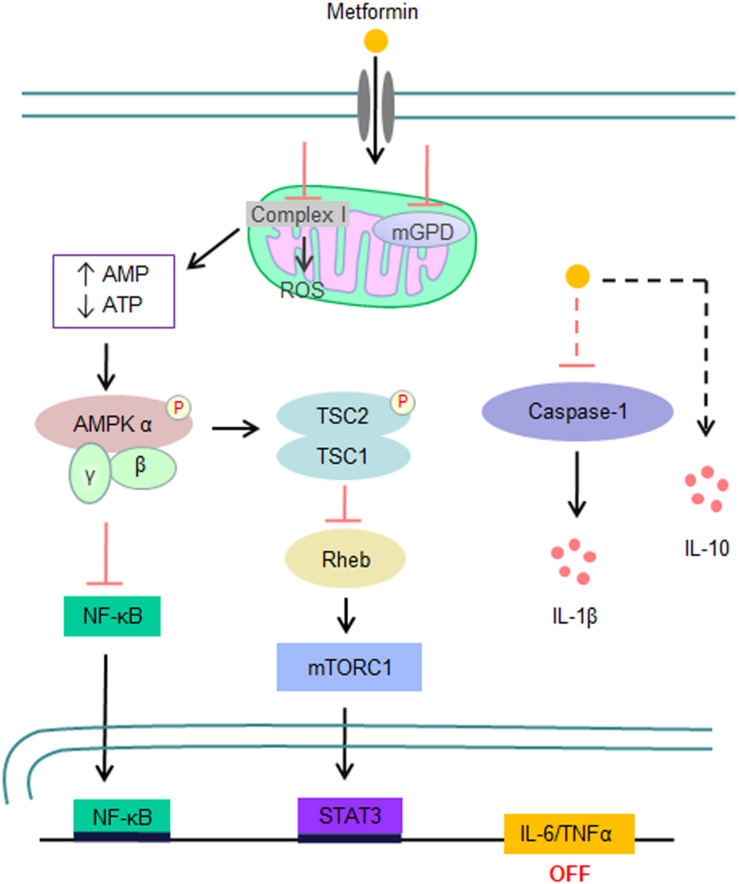
Signaling pathways that are involved in the inhibition of the inflammatory storm by metformin. Metformin directly inhibits complex I activity of the mitochondrial electron transport chain, resulting in an elevated AMP/ATP or ADP/ATP ratio and consequential AMPK activation. Activated AMPK subsequently inhibits the expression of proinflammatory cytokines, such as IL-6 and TNFα, by suppressing the NF-κB and mTOR signaling pathways. In addition, metformin also inhibits IL-1β expression and enhances IL-10 expression by macrophages in response to LPS stimulation in an AMPK-independent manner.

Similar to its glucose-lowering effect, the immunomodulatory effect of metformin mainly depends on the activation of AMP-activated protein kinase (AMPK) (32–34) (Figure 2). In brief, metformin directly inhibits respiratory-chain complex 1 of the mitochondrial electron transport chain, resulting in a reduction in ATP synthesis and thereby an increase in the AMP/ATP or ADP/ATP ratio, which consequentially activates AMPK through the binding of either AMP or ADP. AMPK activation not only switches off mTOR signaling through direct phosphorylation of TSC2 and RAPTOR but also inhibits the NF-κB pathway (31). It has been demonstrated that metformin has an immunosuppressive activity in both *in vitro* and *in vivo* models. For example, metformin inhibits the expression of IL-1β, IL-6, and TNFα by activated macrophages (33, 35–37) but enhances their IL-10 expression (38). Metformin also reduces the release of NETs from neutrophils in patients with diabetes (39, 40). Furthermore, treatment of mice with metformin results in a reduction in the cytokine production of Th1 and Th17 cells and their infiltration in the central nervous system, slowing the disease progression of experimental autoimmune encephalomyelitis (41). Taken together with its suppressive effects on both innate immunity and pathogenic Th cell responses, metformin has the potential to suppress the cytokine storm produced by severe COVID-19.

Studies have shown that metformin alleviates inflammation and protects against acute lung injuries in several animal models. First, metformin treatment significantly reduces lipopolysaccharide (LPS)-induced lung destruction and paraquat poisoning-induced acute lung injury (42–45). Second, metformin attenuates lung injury caused by the high pressure of mechanical ventilation (46). Furthermore, metformin reverses established fibrosis of injured lungs in a bleomycin mouse model (47). Together with the anti-inflammatory effects, these findings support that metformin has the potential to mitigate the inflammation and lung injuries of severe COVID-19 infection.

## Metformin Is a Potential Antiviral Drug for COVID-19

A recent study has identified 332 protein interactions between SARS-CoV-2 proteins and human proteins with affinity purification-mass spectrometry analysis (48). The analysis has revealed that metformin may target the interactions between viral proteins and host factors, such as viral protein Nsp7 and human NDUFA2, and viral protein Orf9c and human NDUFAF1 or NDUFB9, and thus has antiviral activity.

It has also been reported that metformin has antiviral activities in other viral infections via the activation of AMPK (49–52). Metformin inhibits dengue virus infection by restoring AMPK activity (49), which is attenuated at the early stages of dengue virus infection. Similarly, metformin decreases the viral replication of Coxsackievirus B3 (CVB3) and protects mice from CVB3-induced myocarditis, thereby benefiting the survival rate of infected mice (51). In addition to RNA viral infection, metformin also has antiviral activity in DNA viral infections. Metformin treatment *in vitro* drastically inhibits viral gene expression and infectious virion production of Kaposi sarcoma herpesvirus (50). Metformin also inhibits the replication of hepatitis B virus (HBV) in primary human hepatocytes by repressing viral transcription-related genes, including LRH1, PPARα, and HNF4α. Meanwhile, a combination of metformin and entecavir inhibits HBV replication more significantly than either alone (52). These findings suggest that metformin might be used as a potential therapeutic agent for SARS-CoV-2 infection and other viral infections, especially in combination with other antiviral agents.

Furthermore, metformin may also inhibit SARS-CoV-2 infection by interfering with its interaction with ACE2 via the activation of AMPK. AMPK phosphorylates ACE2 Ser680 in human endothelial cells and increases ACE2 expression by enhancing its stability. Metformin also enhances the phosphorylation and expression of ACE2 (53). It is believed that phosphorylation will lead to conformational and functional changes in the ACE2 receptor and decrease the binding of SARS-CoV-2 (54). In addition, ACE2 plays an important role in anti-inflammation and antifibrosis (55). The entry of SARS-CoV-2 into cells by binding to ACE2 downregulates its expression and leads to an imbalance in the renin-angiotensin-aldosterone system (RAS), promoting proinflammatory and profibrotic effects. The imbalance in the RAS is likely averted through upregulation of ACE2 expression by metformin (55). Hence, metformin would not only prevent the entry of SARS-CoV-2 but also decrease its deleterious effects.

Two recent independent retrospective studies have revealed that metformin treatment tends to reduce the mortality of patients with COVID-19 with T2D or obesity (56, 57). One large study revealed that a reduction in mortality is only found in female patients and that sex-dependent survival is associated with a reduction in TNFα.

## Side Effects of Metformin and Routes of Usage

Although metformin is widely used to treat T2D and has a good safety profile, some treated patients develop cutaneous and gastrointestinal (GI) side effects (58–61). Rare cutaneous side effects include leukocytoclastic vasculitis, bullous pemphigoid, lichen planus, and acute alopecia (58). GI side effects are the most frequent adverse effects (59–61) and occur more frequently in patients of older age and in women than in other patients. Approximately 25% of metformin-treated patients develop GI symptoms, including nausea, vomiting, diarrhea, bloating, and abdominal pain (59, 60). As a result, approximately 5% of cases have to discontinue metformin therapy due to intolerant side effects (59, 60). Intolerant patients are mainly older women (59). The GI side effects may result from complications of treatment with other drugs that reduce the function of organic cation transporter 1 (OCT1). In addition, certain OCT1 genotypes are linked to GI intolerance as well. Furthermore, the OCT1 genotypes and usage of OCT1-interacting drugs have a synergistic effect on GI intolerance (59).

In addition to cutaneous and GI side effects, metformin is rarely associated with other adverse effects, such as chest discomfort, heartburn, flatulence, weakness, myalgia, palpitation, flushing, headache, dyspnea, anemia, increased diaphoresis, and lactic acidosis (58). Both lactic acidosis and anemia are rare side effects. Lactic acidosis occurs in patients who have renal dysfunction. Anemia results from the reduction in vitamin B12 levels due to malabsorption (61).

Lowering the starting dose of oral administration with a gradual increase in dosage is recommended to minimize the GI side effects of patients with diabetes. Actually, oral administration is not necessary to treat other diseases. For example, topical administration has been shown to improve histological, clinical, and radiographic outcomes of chronic periodontitis in patients, and no adverse events occur (62). In addition, topical metformin appears to be a safe and effective treatment for melasma (63). Therefore, topical metformin may be suitable for treating inflammation associated with acute lung injury in COVID-19.

## Conclusion and Discussion

Excessive inflammation is involved in the development of severe COVID-19. Inhibition of the inflammatory response might be a promising strategy to mitigate disease severity. Metformin has suppressive activities in the production of proinflammatory cytokines by activated macrophages, the formation of NETs, and the immune responses of pathogenic Th1 and Th17 cells. Studies in animal models have also demonstrated that metformin can alleviate various acute lung injuries. In addition, metformin may directly inhibit SARS-CoV-2 infection by targeting the interaction between human proteins and viral proteins. Furthermore, clinical studies have shown that metformin treatment is associated with a reduction of mortality in diabetic patients with severe COVID-19. Therefore, metformin is promising as a drug candidate to prevent or treat severe COVID-19. Notably, metformin occasionally has adverse side effects, including cutaneous and GI side effects and lactic acidosis. Routes of administration (for example, nebulization) and the synergistic effects with other treatments merit further investigation.

## Author Contributions

QL conceived the presented idea. XC wrote the manuscript and prepared the figures. QL, HG, LQ, QD, and CZ revised the manuscript. All authors contributed to the article and approved the submitted version.

## Conflict of Interest

The authors declare that the research was conducted in the absence of any commercial or financial relationships that could be construed as a potential conflict of interest.
